# Extravascular Implantable Cardioverter-Defibrillator Therapy for Malignant Ventricular Arrhythmias in a Child with Congenital Long QT Syndrome: A Case Report

**DOI:** 10.3390/jcdd13070332

**Published:** 2026-07-15

**Authors:** Xiaodong Sun, Huafeng Wang, Yujia Wang, Fangqi Gong, Liyang Ying, Wei Wang

**Affiliations:** 1 Department of Cardiology, Children’s Hospital, Zhejiang University School of Medicine, Hangzhou 310052, China; 6524131@zju.edu.cn (X.S.); wanghuafeng@zju.edu.cn (H.W.); wangyujia@zju.edu.cn (Y.W.); gongfangqi@zju.edu.cn (F.G.); 2 National Clinical Research Center for Children and Adolescents’ Health and Diseases, Hangzhou 310052, China; yingliyang_1980@zju.edu.cn; 3 Department of Cardiac Surgery, Children’s Hospital, Zhejiang University School of Medicine, Hangzhou 310052, China

**Keywords:** congenital long QT syndrome, torsades de pointes, implantable cardioverter-defibrillator, sudden cardiac death, child

## Abstract

Background/Objectives: Children with congenital long QT syndrome (cLQTS) are at extremely high risk of torsades de pointes (TdP) and sudden cardiac death (SCD). Although the implantable cardioverter-defibrillator (ICD) is a cornerstone in SCD prevention, traditional transvenous ICDs are associated with venous access occupation, lead wear or fracture due to somatic growth, and difficulties with long-term lead revision or extraction. The extravascular ICD, which places the lead in the substernal extravascular space, has the potential to circumvent these lead-related complications. We report the experience of successful extravascular ICD implantation in a young child with drug-refractory cLQTS type 2 (cLQTS2). Methods: The clinical data of a 9-year-old boy with cLQTS2 were retrospectively analyzed. The patient carried a heterozygous KCNH2 variant (c.1810G>A, p.Gly604Ser) and had been on long-term oral propranolol and mexiletine. He presented with a cardiac arrest out of hospital during nocturnal sleep, was transferred to our hospital after successful cardiopulmonary resuscitation, and had TdP captured on ambulatory monitoring. Following multidisciplinary discussion, preoperative chest computed tomography (CT) assessment of the substernal anatomy, and informed consent, extravascular ICD implantation was performed on hospital day 11. The procedure involved the creation of a substernal tunnel via a subxiphoid incision, placement of the defibrillation lead in the anterior mediastinum with lead slack reserved for growth, and positioning of the pulse generator in a left axillary subcutaneous pocket. Intraoperative defibrillation testing succeeded with a single 30 J shock. Results: The postoperative recovery was uneventful without procedure-related complications. At the 2-month follow-up, device parameters were satisfactory and no inappropriate shocks had occurred. The corrected QT interval (QTc) decreased from 563 ms on admission to 522 ms. Ambulatory monitoring detected asymptomatic episodes of non-sustained ventricular tachycardia, but no ventricular fibrillation or syncope was observed. Venous access was fully preserved. Conclusions: In children with drug-refractory cLQTS2, the extravascular ICD provides defibrillation and antitachycardia pacing (ATP) while avoiding transvenous lead complications and preserving venous access. This case shows that with precise preoperative planning and lead redundancy, the device appears feasible and can be implanted without short-term complications in young children. Larger studies with longer follow-up are needed to evaluate long-term device performance.

## 1. Introduction

Congenital long QT syndrome (cLQTS) is a genetic ion channel condition characterized by an extended corrected QT interval (QTc) and torsades de pointes (TdP), which can result in syncope, cardiac arrest, and potentially sudden cardiac death (SCD) [1,2]. β-blocker therapy is the primary treatment for cLQTS. Patients at high risk may also require left cardiac sympathetic denervation (LCSD) or an implantable cardioverter-defibrillator (ICD) [3,4]. ICD implantation is one of the primary preventive interventions for SCD in children with cLQTS. However, traditional transvenous ICD implantation in pediatric patients presents unique obstacles, such as venous access occupation, lead wear or fracture owing to somatic growth, and difficulty with long-term lead revision or extraction. The extravascular ICD, with its lead located in the substernal, extravascular region, offers the potential to avoid lead-related issues. Given the scarcity of experience with extravascular ICD in children, particularly in younger pediatric patients, we present a case of successful extravascular ICD implantation in a 9-year-old boy with cLQTS who showed no complications or inappropriate shocks and had excellent device parameters at follow-up.

## 2. Case Presentation

A 9-year-old boy (height 147.0 cm, weight 34.8 kg, body surface area 1.15 m^2^) with recognized cLQTS type 2 (cLQTS2) suffered a cardiac arrest out of hospital while sleeping at night. Following successful cardiac resuscitation on site, he was brought to our hospital and identified as a high-risk SCD survivor. His hemodynamics were stable upon admission, but electrocardiography revealed a significantly prolonged QTc of 563 ms (Figure 1A). Ambulatory electrocardiographic monitoring revealed a further episode of TdP (Figure 1B), accompanied by numerous polymorphic ventricular premature beats. The cardiac anatomy and function were normal. After admission, intensive antiarrhythmic therapy was initiated, combining oral propranolol and mexiletine, supplemented with potassium and magnesium, as well as measures to maintain emotional stability and a quiet environment. After treatment, the patient had no more convulsions or cardiac arrest, and repeat ambulatory monitoring revealed a significant reduction in ventricular premature beats. Nonetheless, given the cardiac arrest out of hospital, the QTc in a clearly high risk range, and the documented TdP, the patient was deemed to have drug-refractory, extremely high-risk SCD with insufficient long-term protection from pharmacotherapy alone, thus fulfilling a definitive Class I indication for secondary prevention ICD implantation [4].

At the age of 5, the patient was taken to a local hospital due to a sudden loss of consciousness (without cardiac arrest) and was subsequently transferred to our hospital for further treatment. Multiple electrocardiograms showed a prolonged QT interval (maximum QTc 545 ms), ambulatory monitoring revealed multifocal ventricular premature beats and non-sustained ventricular tachycardia, and echocardiography demonstrated normal cardiac structure and function. Genetic testing revealed a heterozygous KCNH2 mutation, c.1810G>A (p.Gly604Ser), which confirmed the diagnosis of cLQTS2. Given the procedural risks, the family initially declined ICD implantation. Prior to the current admission, the patient was on guideline-directed medical therapy, including β-blockers and sodium channel blockers.

The patient’s father and sister were also diagnosed with LQTS. The father died suddenly at the age of 40. The mother is healthy.

Although the patient did not experience recurrent cardiac arrest on standardized pharmacotherapy after admission, the diagnosis of cLQTS2 was unequivocal and the history of cardiac arrest met the Class I indication for ICD therapy in patients with LQTS [4]. LCSD was discussed but postponed, given the recent cardiac arrest and the family’s preference for immediate secondary prevention with a device. Preoperative chest computed tomography (CT) was performed to precisely assess the substernal anatomy (Figure 2) and to meticulously plan the electrode implantation pathway. After a multidisciplinary team discussion, reevaluation of the procedural risk–benefit ratio, thorough communication with the parents, and obtaining informed consent, the patient underwent extravascular ICD implantation on hospital day 11.

Under general anesthesia, the extravascular ICD defibrillation lead was advanced through a subxiphoid incision and along the substernal tunnel. The procedure was adapted to the pediatric thoracic anatomy: after blunt dissection through the subxiphoid space into the retrosternal region, the tunneling tool was advanced closely against the posterior sternal wall, and lead position was confirmed by fluoroscopy within the cardiac silhouette. During lead insertion, the blue indicator band was maintained at the hemostatic valve as per standard protocol to leave sufficient lead slack for future growth. The pulse generator was placed in a subcutaneous pocket near the left anterior axillary line and connected to the lead via a subcutaneous tunnel. Intraoperative defibrillation testing demonstrated appropriate sensing and successful conversion of ventricular fibrillation with a single 30 J shock. The key intraoperative device parameters are summarized in Table 1. Postoperative chest radiography confirmed satisfactory lead and generator position (Figure 3).

The patient was discharged in stable condition postoperatively. Long-term post-discharge management focused on reducing the risk of recurrent cardiac arrest. On a foundation of intensified antiarrhythmic therapy with propranolol and mexiletine, continuous oral potassium and magnesium supplementation was maintained to stabilize myocardial electrical activity. Strict avoidance of sympathetic triggers known to provoke TdP, including emotional excitement and sudden awakening from sleep, was emphasized. A regular schedule of device and clinical follow-up was established to periodically interrogate extravascular ICD parameters, monitor for inappropriate shocks, and dynamically assess QTc, electrolytes, and cardiac function.

At the 2-month follow-up visit, no complications or inappropriate shocks were observed, and device parameters were satisfactory. Electrocardiography showed a QTc of 522 ms. There were asymptomatic episodes of non-sustained ventricular tachycardia, but no ventricular fibrillation or syncope occurred.

## 3. Discussion

cLQTS is a hereditary cardiac disease caused by mutations in cardiac ion channel genes. This case involves cLQTS2 resulting from a KCNH2 mutation, which leads to loss of function of the Kv11.1 potassium channel and QT interval prolongation [5]. Arrhythmic events in cLQTS2 are frequently triggered by sudden startles, such as auditory stimuli, and it has been reported that approximately 29% of events occur at rest or during sleep [6]. In the present case, the child experienced a sudden cardiac arrest during sleep and was successfully resuscitated before transfer to our hospital, consistent with the typical triggers and circumstances of cLQTS2 described in the literature. Furthermore, a recent large cohort study of children and adolescents with cLQTS in Germany confirmed that cLQTS2, a QTc > 500 ms, and a history of aborted cardiac arrest are core risk factors for malignant cardiac events in pediatric patients [2].

Regarding pharmacotherapy, β-blockers are the first-line treatment for cLQTS, and high-dose propranolol or nadolol has clear efficacy in cLQTS2 [4]. Mexiletine, a sodium channel blocker, can also shorten the QTc and exert antiarrhythmic effects, and is often used as an add-on therapy [7]. After admission, an intensive antiarrhythmic regimen was initiated, combining oral propranolol and mexiletine supplemented with potassium and magnesium. Following treatment, ambulatory electrocardiography showed a marked reduction in ventricular premature beats, and clinical symptoms improved. However, while on adequate doses of propranolol and mexiletine, the patient experienced a cardiac arrest out of hospital during sleep, and ambulatory monitoring additionally captured an episode of TdP, clearly indicating the limited protective effect of pharmacotherapy alone and placing the patient at high risk for drug-refractory SCD. According to the 2022 ESC guidelines [4], survivors of cardiac arrest with LQTS are recommended to receive ICD implantation in addition to oral β-blockers (Class I recommendation). The 2021 PACES expert consensus [8] specifically highlights that for pediatric patients expected to require lifelong ICD therapy, the choice of device type must fully consider the preservation of vascular access, long-term lead durability, and the cumulative risks of multiple replacements. This patient is a cardiac arrest survivor, fully meeting the Class I recommendation, and has a definite indication for secondary prevention ICD implantation.

With regard to device selection, for a 9-year-old child, it is crucial to choose a device that can both effectively prevent SCD and minimize long-term iatrogenic risks. Traditional transvenous ICDs provide defibrillation, antitachycardia pacing (ATP), and long-term bradycardia pacing. Among these, increasing the basic heart rate to avoid the “vulnerable window” may have a preventive effect on some rate-dependent malignant arrhythmias [9,10]. However, the antiarrhythmic efficacy of high-rate pacing is highly dependent on the specific etiology and electrophysiological substrate. In certain pathological states such as heart failure, it may even aggravate repolarization heterogeneity [11], and large-scale clinical evidence in children is lacking. More importantly, this patient had no sustained bradycardia and no clear indication for pacing. Trading unconfirmed long-term pacing benefits for the lifelong cumulative risks inherent to transvenous leads, including venous thrombosis or stenosis, tricuspid regurgitation, lead fracture, infection, and multiple replacements, is unreasonable for a 9 year old. Studies have shown that the complication rate of transvenous ICD in young patients can be as high as 21~41% [12]. The PLEASE study further confirmed that the annual lead failure rate in children and young patients is substantial, and the difficulty of lead extraction is positively correlated with implant duration [13]. More recently, Hong et al. [14] demonstrated that greater somatic growth is an important predictor of lead failure in pediatric patients, with growth exceeding 5 cm per year associated with a more than 3-fold increase in the risk of lead failure (HR 3.33; 95% CI: 1.78–6.25). Furthermore, although the entirely subcutaneous ICD avoids intravascular leads, it cannot provide ATP therapy [15]. This patient had documented TdP and frequent polymorphic ventricular premature beats; ATP is clinically valuable for terminating some ventricular tachycardias and reducing shock burden. Therefore, a subcutaneous ICD without ATP capability is not the optimal choice. Recent reports have indicated that subcutaneous ICD has a higher rate of inappropriate shocks in young patients with channelopathies, partly due to limited discrimination of T-wave oversensing [16]. In a cohort of teenagers and young adults with subcutaneous ICDs, Bettin et al. [17] reported an inappropriate shock rate of 16.1%, with all events occurring in patients under 20 years of age, and identified younger age as an independent predictor. Because ATP is unavailable, every arrhythmic event must be treated with a shock.

The extravascular ICD places the defibrillation lead in the substernal, extravascular space, providing defibrillation and ATP while eliminating transvenous lead-related complications by design and completely preserving venous access. For pediatric patients with decades of expected survival, preserving venous access is of significant long-term clinical importance. Once venous occlusion occurs due to a transvenous lead, future options such as pacemaker upgrades, central venous catheterization, and even hemodialysis can be severely restricted [18]. The device is relatively small, with an estimated service life of approximately 11.7 years. In the Pivotal study [19], the extravascular ICD achieved a 100% defibrillation success rate for spontaneous ventricular arrhythmias and an ATP success rate of approximately 77% for terminating ventricular tachycardia. The rate of freedom from major system or surgical complications at 3 years was 89.0%, and overall efficacy was comparable to that of transvenous ICDs. In a subgroup analysis of patients with ion channel diseases in the Pivotal study, those with LQTS and similar conditions had arrhythmia conversion success rates and ATP efficacy consistent with the overall population, with no specific complications [19]. In contrast, multiple studies have reported that subcutaneous ICDs, lacking ATP, face a higher risk of inappropriate shocks in young patients [16,17]. This functional difference, combined with the extravascular ICD’s sensing and discrimination algorithms that are more similar to traditional ICDs, could theoretically result in a lower shock burden for young patients. In terms of pediatric application, Spentzou et al. [20] demonstrated that extravascular ICD implantation in adolescents and low-weight children has a high success rate, reliable ATP efficacy, and good short-term safety. A recent single-center study of extravascular ICD implantation in pediatric patients showed that the substernal lead pathway is anatomically feasible in children weighing as little as 30 kg, with a high procedural success rate, and that adequate lead slack can be reserved during surgery according to the child’s thoracic dimensions to accommodate growth [21]. Furthermore, a case of successful extravascular ICD implantation in a 2-year-old child has been reported [22], further lowering the age and weight boundaries of the technology. For this patient, the extravascular ICD precisely met the multiple requirements of effective SCD prevention, ATP capability, preservation of vascular access, and minimized long-term lead risks. Therefore, our center chose the extravascular ICD over a transvenous ICD and subcutaneous ICD. Short-term follow-up showed stable device parameters, no inappropriate shocks, and no procedure-related complications, preliminarily confirming the safety and efficacy of this approach in a young child with cLQTS2.

The published literature on pediatric extravascular ICD implantation remains limited. Spentzou et al. [20] reported a UK single-center initial experience of extravascular ICD implantation in children, including a 6-year-old patient with LQTS weighing 20 kg, and emphasized the importance of preoperative cross-sectional imaging for procedural planning, particularly in younger children. In the single-center studies by Walsh et al. [21] and Dragisic et al. [23], the median ages at implantation were 14.5 and 16.8 years, respectively. Walsh et al. [21] reported a minimum weight of 27 kg, with pleural lead placement in that patient due to difficult diaphragmatic entry. The youngest reported case is a 2-year-old child [22], but that case did not involve LQTS, and body weight was not provided. In the present case, the preoperative CT scan measured a substernal space of 5.90 mm at a body weight of 34.8 kg, which permitted safe lead passage. Whether this technique can be safely performed in children weighing 25 kg or less remains to be determined, given that technical challenges such as difficult diaphragmatic entry have been reported at the lower end of this weight range in published pediatric series. Overall, there is a gap in the literature regarding low-age children with cLQTS2, particularly those with detailed descriptions of the device decision-making process in the context of drug breakthroughs. This case thus provides complete clinical experience of successful extravascular ICD implantation in a 9-year-old child with cLQTS2 following the failure of adequate β-blocker and mexiletine therapy. In particular, it demonstrates a feasible pathway for preoperative evaluation, intraoperative tunnel creation, and parameter testing under the anatomical constraints of a smaller thorax requiring lead slack for growth. This experience thereby enriches the clinical evidence for device therapy in this special population.

This study has certain limitations: experience with extravascular ICD in children is limited, the follow-up period in this case is short, and problems related to the child’s growth, lead migration, or P-wave oversensing may appear months or years after implantation. The long-term durability of the device beyond 10 years in this low-age population requires continued observation. Therefore, the safety conclusions are preliminary and based on short-term follow-up only. Nonetheless, this case provides a clinical practice reference for device selection in drug-refractory low-age children with cLQTS2, suggesting that after carefully balancing long-term risks, the extravascular ICD can offer an alternative strategy for SCD prevention in such patients.

## 4. Conclusions

The extravascular ICD achieves dual therapy with defibrillation and ATP via a completely extravascular approach, providing an effective strategy for treating malignant arrhythmias in high-risk children with cLQTS2 caused by KCNH2 mutations. Our experience suggests that with meticulous preoperative anatomical assessment and intraoperative provision of lead slack for growth, this device appears feasible and can be implanted without short-term complications in young children with a small thorax. Future studies with longer follow-up and larger pediatric cohorts are needed to evaluate the long-term evolution of sensing performance and the durability of the system beyond 10 years in this low-age population.

## Figures and Tables

**Figure 1:**
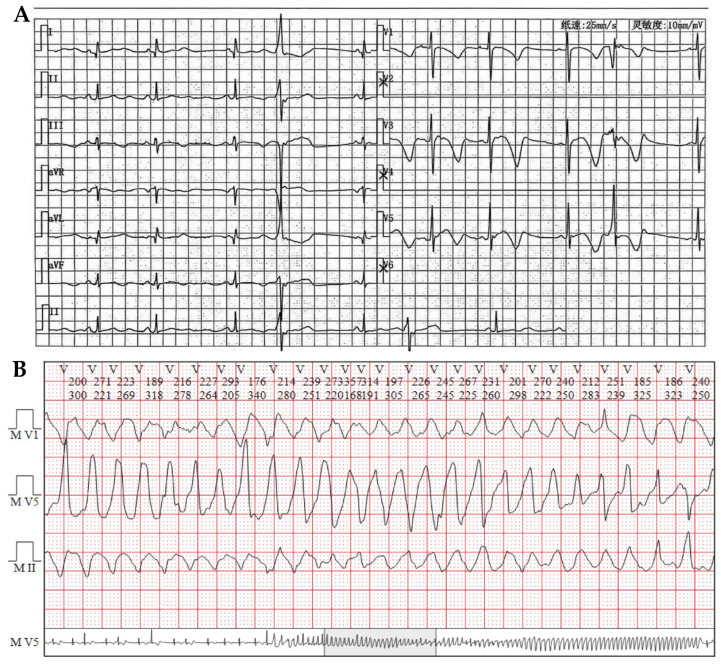
Electrocardiogram and ambulatory electrocardiographic monitoring on the day of admission. (**A**) Electrocardiogram on admission demonstrating a QTc of 563 ms. Paper speed: 25 mm/s, Sensitivity: 10 mm/mV. (**B**) Ambulatory electrocardiographic monitoring on admission captured an episode of TdP.

**Figure 2:**
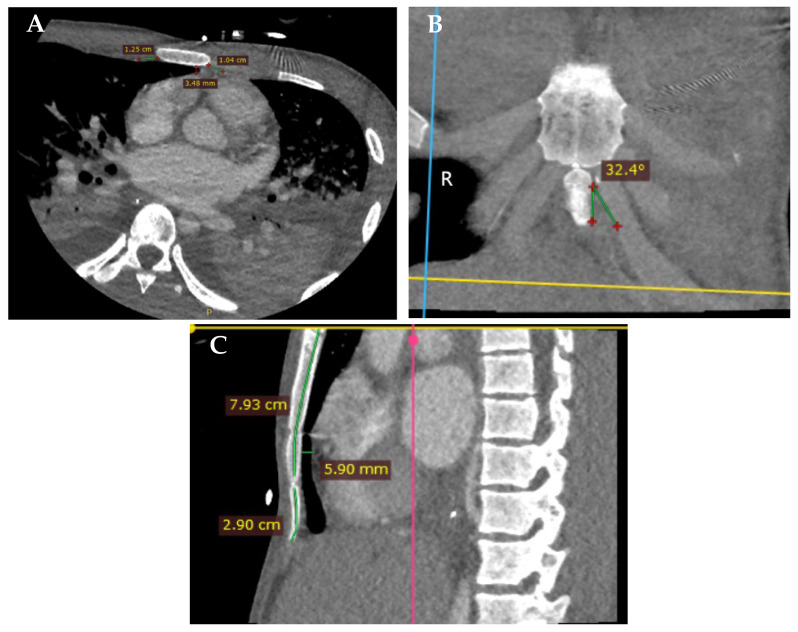
Preoperative chest CT for precise evaluation of the substernal anatomy. (**A**) The distance from the internal mammary artery to the sternum ranged from 1.04 to 1.25 cm. (**B**) The xiphoid process was normally developed. (**C**) The sternum was straight. Red crosses mark the measurement anchors. Green lines represent the measured length and angle values. “R” and “P” denote the right and posterior anatomical orientations. The yellow, blue, and pink reference lines indicate the axial, coronal, and sagittal planes.

**Figure 3:**
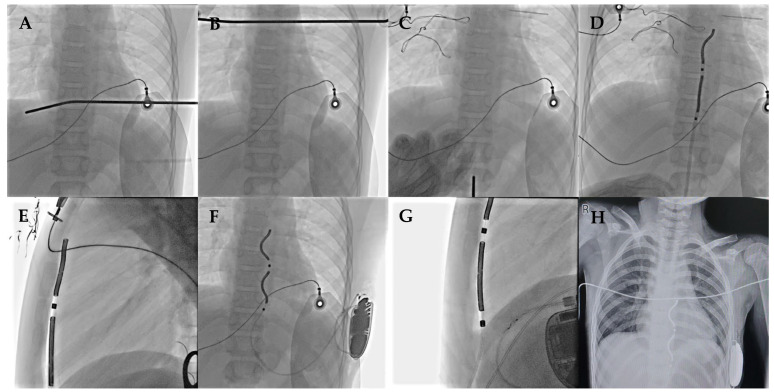
Intraoperative imaging of the extravascular ICD implantation procedure and a postoperative posteroanterior chest radiograph. (**A**) Creation of the substernal tunnel via the subxiphoid incision under fluoroscopic guidance. (**B**) Positioning of the tunneling tool against the posterior sternal wall. (**C**) Delivery of the defibrillation lead through the introducer sheath. (**D**) Anteroposterior fluoroscopic view confirming that the lead is oriented vertically along the left sternal border. (**E**) Lateral fluoroscopic view assessing lead depth and apposition. (**F**) Connection of the pulse generator within the subcutaneous pocket. (**G**) Lateral fluoroscopic view verifying stable lead course. (**H**) Postoperative posteroanterior chest radiograph demonstrating correct position of the system. “R” denotes the right anatomical side of the patient.

**Table 1:** Intraoperative device parameters at implantation.

Parameter	Value
Baseline R-wave amplitude	3.9 mV
Defibrillation testing	30 J, successful
Pacing threshold	4.0 V
High-voltage shock impedance	55 Ω
Pacing impedance	285 Ω
Programmed VF zone	194 bpm (310 ms), On
Programmed VT zone	167 bpm (360 ms), On
SVT discrimination features	Enabled (Wavelet, Rapid AF, Feature Match)

VF: ventricular fibrillation; VT: ventricular tachycardia; SVT: supraventricular tachycardia; AF: atrial fibrillation.

## Data Availability

Further anonymized data related to this study are available from the corresponding author upon reasonable request.

## Abbreviations

The following abbreviations are used in this manuscript.

cLQTS	congenital long QT syndrome
TdP	torsades de pointes
SCD	sudden cardiac death
ICD	implantable cardioverter-defibrillator
CT	computed tomography
QTc	corrected QT interval
ATP	antitachycardia pacing
LCSD	left cardiac sympathetic denervation
